# Response of Fattening Rabbits with Acorns (*Quercus pubescens* Willd.) Combined in the Diet: First Acquaintances on Growth Performance, Carcass Traits and Perirenal Fatty Acid Profile

**DOI:** 10.3390/ani10081394

**Published:** 2020-08-11

**Authors:** Petra Wolf, Maria Grazia Cappai

**Affiliations:** 1 Institute of Nutrition Physiology and Animal Nutrition, University of Rostock, Justus-von-Liebig-Weg 6b, 18059 Rostock, Germany; 2 Department of Veterinary Medicine, Università di Sassari, Via Vienna 2, 07100 Sassari, Italy; mgcappai@uniss.it

**Keywords:** fatty acids, flavour, functional food, PUFA, rabbit

## Abstract

**Simple Summary:**

Consumption of rabbit meat has increased markedly over the last 50 years. This trend appears to be a driving force behind modern farming practices, in particular of rabbit feeding, more and more oriented to fulfil consumers’ demand of sustainable, welfare and health-friendly and low-impact animal production. In this context, the deployment of alternative feeding sources with particular biological properties in the production of feed for meat-producing animals is worthy of being investigated. This trial explored the effect of the combination of acorns as a whole ingredient in the diet of fattening rabbits with the aim to acquire pioneering information on the production and health parameters, in view of the potential effects of the diet on growth, carcass wear and fatty acid composition of perirenal fat.

**Abstract:**

The request for functional and healthy meat presents a challenge to modern animal nutritionists and rabbit meat consumption appears to increase alongside the aging population. Novel functional feeds for food-producing animals gather the interest of the scientific community and acorns appear frequently accounted among non-competitive-with-human feeding sources, above all in slow food production systems. This investigation aimed to assess the response to acorns combined in the diet of 40 fattening rabbits, in respect of growth performance, carcass characteristics and fatty acids composition in perirenal fat. A same commercial fattening diet combined or not with shredded acorns (control, CON = 0 vs. acorn combined diet, ACD = 200 g/kg feed as fed weight, respectively) was administered for six weeks to two groups of Separator rabbits, consisting of 20 animals each. No differences in feed conversion, carcass weight at slaughter and carcass yields (24 h) were found between groups at the end of the experimental feeding. Perirenal fat profile of rabbits from the ACD group pointed to significant differences in ΣPUFA content (25.1 vs. 31.6, as a percentage of total lipids, respectively, *p* < 0.001) and in the Σ *n* − 6/*n* − 3 ratio (5.95 vs. 2.41). In conclusion, acorns can be used as an energy source in mixed feeds for rabbits, especially in slow production systems.

## 1. Introduction

Consumption of rabbit meat increased markedly over the last 50 years and this trend appears to be shaping modern farming practices. In particular, feeding practices are oriented to fulfil consumers’ demand of functional and healthy meat, and for ethical and sustainable food production [1]. Recently, the potential of rabbit meat as a functional food was reviewed [2] in light of the most up-to-date breeding and feeding strategies. In this scenario, the increasing aging population and the high prevalence of metabolic disorders represent public health issues in developed countries, which seem to positively correlate and drive consumer′s preference for rabbit meat. Such a demand poses a question to animal nutritionists about the chance offered by functional feedstuffs to be used in rabbit feeding practices of slow production systems. In this regard, investigations on biologically active compounds, purified or contained in alternative ingredients used in dietary formulations, continuously gather the interest of the scientific community in the field of animal nutrition, as well as of feed and food industries. In some Mediterranean countries, different extensive animal productions take advantage of the seasonal availability of natural feeding stuffs, which turned to be fundamental ingredients for traditional meat productions as a non-competitive-with-human feeding source. The use of ripe acorns for grazing and browsing livestock in oak forests appears a valuable seasonal feeding strategy thanks to energy concentration, polyphenolic profile, Vitamin E and high Zn contents [3,4,5,6,7,8,9,10,11,12]. Acorns can be consumed directly by free ranging animals or else be included in mashed diets, should free grazing not be practicable [7]. In view of this aspect, acorns can also be collected and offered to provide sustainable dietary energy sources to semi-extensively raised pigs during the fattening period, especially for the production of cured ham, thanks to high starch and a peculiar acidic profile [13,14].

Nutrient composition of acorns identifies these fruits as energy-rich feedstuffs (starch 512–571 g and 42–63 g crude fat content in kg dry matter) [14]. Therefore, acorns can serve as concentrate feeds in extensive breeding systems [14] chiefly in feed mixtures [15,16] due to biologically active compounds (polyphenols profile) and to the peculiar pattern of fatty acids [14]. A number of studies on the use of acorns was carried out in pigs and, to a lesser extent, in ruminants and poultry. Although the study by Zamora-Lozano and co-workers [17] provided very promising results, only a few studies in rabbits can be found in the scientific literature. An advantage of the use of acorns in animal feeding resides in the production of special niche products, like in the case of several well-known pork productions (Iberic pork and its Iberic ham or Nebrodi pork production, for instance [18,19]).

Acorns are rich in polyphenols and their biological role as natural anti-oxidants may also be exploited to confer stability to rabbit meat. However, acorns are known to be rich in hydrolysable tannins, varying in spectrum and concentration according to the botanical origin and ripening of the fruit. At his regard, it is well known that some animal species are capable of tolerating acorn tannins in the diet [20] whilst others are not. Such adaptation to cope with tannin-rich feeding stuffs is, in some cases, mitigated by salivary secretion and composition. In particular, parotid gland secretions are called forth when potentially toxic substances of diverse origin are ingested [21]. The success to buffer the protein precipitating activity (PPA) of tannic acid (TA) is due to the secretion of tannin binding proteins, in particular histatins and proline-rich protein (PRP) [22]. Against this background, basic data on the safe use of acorns as functional feeds in rabbits appears scanty to date. In view of this state of the art, the question on the use of acorns as functional ingredients in the diet of fattening rabbits needs to be elucidated. For this reason, a feeding trial was carried out with the aim to explore the effects of acorn inclusion in the diet of fattening rabbits and to correlate this ingredient provision with production performance in vivo as well as with carcass characteristics. In addition, first acquaintances would be acquired about the meat flavour and fatty acid profile of perirenal fat as a body source to evaluate the change of animal fatty acid composition in view of acorn intake.

## 2. Materials and Methods

### 2.1. Animals and Diets

Animal handling complied with the recommendations of European Union Directive 2010/63/EU [23] concerning animal care. All procedures reported in this trial belong to conventional clinical and breeding practices; in particular, feeding, body weight record and slaughter were carried out in respect of current European legislation on animal protection. The study was approved by the State Office for Agriculture, Food Safety and Fisheries of Mecklenburg-Western Pomerania, no. 7221.3-3.2-002/17.

The experiment was carried out at the University of Rostock, enrolling 40 weaned, 36 days-old rabbits (20 female/20 male rabbits, breed: Separator; body weight at start: 703 ± 22.5 g). Animals came from one same farm and were distributed to two groups according to a matched-pairs approach, based on body weight and litter origin (no. 8 litters from relative does), in respect of a sex ratio 1:1 in both groups. Each group consisted of 20 rabbits. All animals were individually housed in metabolic cages (90 × 90 cm, 8100 cm^2^) without bedding material. During the following 40 days, one groups was fed a commercial fattening feed, whereas the other group was fed the same commercial fattening feed combined with 200 g of shredded acorns (collected in Sardinia and belonging to the *Q. pubescens* Willd. oaks) per kg diet, on an as fed basis (see Table 1), later included in the pellet. Feed was offered ad libitum (calculated according to the maximum daily DM intake in g as a percentage of body weight and adjusted according to daily consumption) and all the animals had free access to fresh water throughout the entire trial. Experimental diets were conceived according to the ingredient combination technique [20], which aims to provide information on the effect of a whole ingredient at a certain amount in the diet. In this experiment, acorns were used to substitute sunflower extruded meal and inclusion amount tested was established to explore whether a safe tannic acid intake can be tolerated in relation to rabbit health and response. For these purposes, acorns were introduced in the pelleted feed (20% in the experimental diet, as fed). Moreover, acorns were previously characterized for the full polyphenolic spectrum in previous trials [24]. Feed intake of rabbits in both groups was determined daily, whereas body weight was recorded once a week for average daily weight gain calculation. Feed leftover was removed from the feeder and weighed daily. On the basis of weight and chemical composition of feed and leftovers, determinations were used to calculate daily DM and tannin acid equivalent (TAE) intakes per kg BW.

### 2.2. Chemical Analyses

The chemical composition of the diet and samples was carried out according to proximate analysis, and modified methods [25], in duplicates of each sample. Starch content of the diets was determined according to the official polarimetric method (1999/79/CE) [26]. From whole acorn samples, different aliquots were used to carry out further chemical analyses, like amino acids (AA, in particular lysine, cysteine, methionine and proline) were determined by ion-exchange chromatography (amino acids analyser: Eppendorf-Biotronic, model LC 3000, Eppendorf.Biotronic, Hamburg, Germany). Macro and micro elements were determined by atomic absorption spectrometry except for P. The total polyphenols and the TAE were determined by the Folin–Ciocalteau [27] modified method [28]. Analysed nutrient contents of the two experimental diets are reported in Table 2. A sample of crude fat was methylated to obtain the fatty acid methyl esters (FAME): thereafter, FAME were analysed using a Hewlett–Packard HP-5890 gas chromatograph (Agilent Technologies Inc., Lexington, MA, USA) and identified through standard samples. The content of polyunsaturated fatty acid (PUFA) in DM was determined and expressed on total crude fat. The fatty acid profile of the two experimental diets is reported in Table 2.

### 2.3. Response of Parotid Glands to Dietary Acorn Tannins, Quantitative Evaluation of the Carcass and Organoleptic Traits of Meat

At the end of the experiment (day 76) all animals were slaughtered in a conventional slaughterhouse, where the carcasses were tracked in the slaughtering chain and were subjected to both carcass evaluation and organoleptic testing (roast test).

The assessment of morphology and composition of the parotid gland (PG) was carried out as a way to estimate rabbit response to dietary tannins contained in the acorn combined diet, in the attempt to counteract the protein precipitating activity of TAE. The evaluation of the PG followed the method described by Cappai and co-workers [9,20] in pigs. Briefly, both PGs were surgically removed from the carcass of each rabbit, and excised in toto. After removing the separable fat, lymph nodes and associated vessels, each PG was immediately weighed on a digital scale. Length and width (mm) were measured by means of an electronic digital caliper (799A-6”/150 mm, L.S. Starrett Company, Athol, MA, USA).

The glands in toto were oven dried (103 °C) and then ground; samples were analysed in duplicate for dry matter, crude protein and amino acids as described earlier. Proline content on the dry matter of the respective parotid gland was calculated as a response parameter for proline rich protein synthesis. Relative fresh weight of each parotid gland was calculated according to the body weight of the respective animal before slaughter and expressed as percentage. A proline/CP ratio was also calculated based on proline content (g/kg DM) and CP content (g/kg DM) of the same parotid gland.

For the organoleptic test, an aliquot of 5 × 5 × 1 cm was extracted out of the musculus quadriceps femoris. In brief, on each warm carcass, a surgical window at the level of the proximal third of the lateral side of the left leg was open by means of a scalpel with single use blade and 1 cm thick muscle sample removed. Each muscle sample was stored in individual falcon tubes (50 mL) to be transported to the laboratory in a freezing bag. For the taste panel only meat flavour was assessed at this stage. Each muscle sample was roasted in a pan with corn oil at constant temperature and time, following internal standard method of the laboratory. The samples were tested by two trained persons (always the same ones) who judged whether the meat was more or less aromatic though a semi-quantitative evaluation score (1–5 point scale, where 1 is less aromatic and 5 is for very aromatic). This worked as a preliminary assessment due to the presence of natural tannic acid (expressed as TAE in acorns, known to be a flavouring agent.

The determinations of lipid and fatty acid profiles were carried out on perirenal fat. This evaluation was driven by the establishment that in rabbits’ perirenal fat depot strongly correlates with all carcass fat weight [29]. Kidneys were excised from each warm carcass and separable fat removed and stored in an individual fashion to be transported to the laboratory. Samples were processed for fatty acids following the methods reported above.

### 2.4. Calculations and Statistics

For the evaluation of the normal distribution, the *t*-test for paired observations was used. Data were analysed with a general linear model as reported below:

Y*_i,j_* = *μ* + D*_i,j_* + Z*_m,n_* + D × Z + e*_i,j,m,n_* (1)

where Y is the effect on PG response, sensory score and fatty acids profile, *μ* is the overall mean, D is the fixed effect of the diet (two levels: 0 vs. 20% of acorns in the diet), Z is the fixed effect of gender, D × Z is the interaction between the diet and gender and *e* is the random error. Data were analysed by means of Minitab_18 (^©^2020 Minitab, LLC, State College, PA, USA). All statements of statistical significance are based upon *p* < 0.05.

## 3. Results

No differences in the daily amounts of feed consumed were observed between groups (see Table 3). No differences between genders were observed.

The TAE of acorns were 51.6 g/kg DM providing an amount of 10.2 g TAE/kg DM in the combined diet. Salivary glands (PG) of the rabbits fed with the acorn combined diet displayed significant differences in response to the tannin-rich acorn-based diet, as to increased proline content as well as to the weight and size of the gland (Table 3).

Table 4 reports the fatty acid profile of perirenal fat of rabbits from both groups. Significantly, PUFA content is higher in the perirenal fat of rabbits fed with the acorn combined diet. In addition, Σ n-6/Σ n-3 ratio in acorn fed rabbits displayed to be significantly different (Table 4).

**Figure 1:**
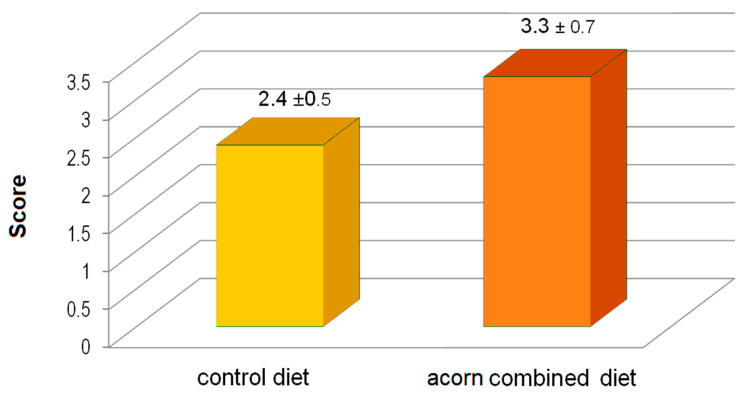
Histograms of scores (1–5 point scale) of a panel test of roasted meat from musculus quadriceps femoris assessed for aromatic properties by independent trained experts. Values are expressed as mean values ± standard error.

The flavour of rabbit meat revealed differences between the two groups fed with the different diets. In fact, in the 94.7% of tests the meat from rabbits fed acorns was assessed to be more aromatic than that of rabbits from the control group (Figure 1). This finding was not correlated with the fatness of the carcass and, in turn, with the energy content of the ration.

## 4. Discussion

This feeding trial was carried out to shed a new light on the potential use of acorns in the diet of fattening rabbits. The investigation was planned to explore in vivo and post mortem aspects, on both production and physiological sides. In fact, the tested amount of acorn inclusion (200 g/kg, as fed) offered on a daily basis to rabbits during the fattening period established the first information on the daily intake of the acorn-based diet, in comparison with the control diet. Results of calculations on the daily weights of feed offered minus left-overs did not point to differences in the average feed intake between groups (data not shown). Live body weight collected throughout the trial until slaughter of rabbits pointed to similar daily gains and carcass weight. Interestingly, despite production performance (feed conversion ratio, final body weight) in vivo, we did not highlight differences between groups, significant post mortem differences were observed if rabbits were fed with the acorn-based diet. The hypertrophy of the parotid gland point to the tolerance of the tannin-rich diet by rabbits. Rabbits of the acorn combined diet group showed a significantly higher weight of the gland as well as proline content, at tested amounts. The presence of polyphenols with protein precipitating activity expressed as TAE contributes to forming complexes in the gastrointestinal content between dietary and endogenous proteins from digestive secretion and microbial synthesis which are rich in non-essential amino acids (e.g., proline). In agreement with what was observed in parotid glands of pigs fed with acorn combined diets [9,20], and salivary glands of other domestic and wild animal species [22], as well as in primates [30,31], the response to TAE can take advantage of salivary production of proline-rich proteins (PRPs), representing a first mechanism of defence against the protein precipitating activity of dietary tannins, like in this case. This mechanism of defence against dietary tannins could be observed in other animal species adapted to tannin-rich diets with the aim to contrast the protein precipitating activity (PPA). This finding is suggestive of the fact that the adaptation to tannin-rich diets in the rabbit should be efficacious, given the modulation of the secreting activity of the PG from augmented synthesis of proline-rich proteins (PRPs) in the saliva and other potential secretions across the gastrointestinal tract. Such a response involving salivary composition is known to be genetically encoded. In view of being capable of modulating the salivary secretion in response to the PPA of dietary tannins, rabbits may appear tolerant to tannin-rich diets. The presence of tannins and the natural AA pattern of acorns may noticeably reduce the availability of essential AA, with particular regard to sulphur amino acids (SAA). However, no significant depression of daily feed intake was observed in rabbits fed the acorn combined diet in comparison with rabbits fed the control diet in this feeding trial. On the other hand, the favourable pattern of fatty acids of acorns, especially oleic acid, may cover a significant role in the stability of rabbit meat, though not explored in this investigation. To such an extent, the desired effect from acorn consumption observed in pigs appears to also be obtained in rabbits enrolled in this trial, in terms of safe use and potential effect on fatty acid profile of perirenal fat.

In both groups, similar carcass weights were observed, which are also common in animals of approximately the same size [32,33,34]. By mixing acorns in the diet, the energy content of feed decreased [35,36]. The protein content in the compound feed decreased, with the amino acids lysine, methionine, cystine and proline, in particular, being lower in the experimental group receiving the acorn combined diet (see Table 2). This can be attributed to the lower protein content of acorns, which, according to Galvan and co-workers [37], varies in magnitude between 29 and 59 g/kg. The protein content of 57 g/kg analysed in this study corresponds to the upper value of the range from the literature, but also coincides with values from other studies, of 59 [38] and 56 g/kg [39]. Cappai and co-workers [14] reported an average value of 37.1 g/kg. This finding may also depend on the botanic origin of acorns which display varying nutrient contents according to genetic type of oak acorns, also involving TAE content [16]. In all cases, acorn feeding at tested amounts during the fattening period of rabbits points to the safe and sustainable use of acorns as feed ingredients, highlighting the potential of employment as alternative feed in slow production systems. It is also to point out that no economic evaluation has been carried out on the use of acorns to feed fattening rabbits at present, because this practice needs to be explored further. However, results obtained from this investigation appear encouraging and pave the way to future investigations oriented to characterize meat quality traits. In this context, trials are currently ongoing with the final aim to protect the origin of a future local product branded “Mecklenburger Oak Rabbit”.

Worth noting, results on the acidic profile of perirenal fat and meat sensory properties highlight significant differences between groups. In particular, the effect on the fatty acid profile from acorn intake is well-described in pigs [11,35,36,40]. No descriptions about the fatty acid profile in rabbit fat from acorn consumption are available in the literature to the best of our knowledge. The sensory properties of meat point to different flavours if rabbits are fed with acorns, likely also due to natural TAE in acorns. These results, although preliminary, pave the way to further investigations aimed to explore the effect of acorns in the diet of rabbits for meat quality trait assessment. Additionally, acorns are also valuable sources of tocopherol, involved in several biological activities within the animal body and, in particular, capable to confer a certain stability against the oxidation of meat [11,12]. Like that observed in other herbivores and, in particular, in hindgut fermenters [41,42] Vitamin E status is strictly related to dietary intakes. Deficiency of dietary tocopherols are described in the literature to cause health disorders in rabbits [43]. The provision of tocopherols through acorn feeding may contribute to improving rabbit meat stability, as well as support overall health by helping systemic antioxidant status. However, this aspect was not studied in this trial, but represents the focus of future studies in this direction, with the objective to better characterize the effects on production performance of acorn feeding in rabbits.

## 5. Conclusions

This preliminary study shows that acorns can be safely used in fattening rabbits at tested amounts. Rabbits were able to respond successfully to the presence of hydrolysable tannins (TAE) with protein precipitating activity in the diet, naturally contained in acorns, by increasing parotid glands, likely mirroring the augmented secretion of PRPs. Perirenal fat depot was not affected by the diet but fatty acid profile displayed to change: this aspect can allow to consider the potential effects of acorn feeding on the fatty acid profile of meat for future studies. The encouraging results focusing on acorns combined in the diet of fattening rabbits pave the way for further investigations to characterize the meat quality and profile of acorn-fed rabbits as an alternative feeding practice and sustainable management of local rabbit production systems.

## Figures and Tables

**Table 1:** Analysed nutrient composition of pure acorns.

Nutrient	Unit	Content
Dry matter	g/kg as fed	612
Crude ash	g/kg DM	21
Crude protein	g/kg DM	57
Crude fat *	g/kg DM	83
Crude fibre	g/kg DM	1445
NfE	g/kg DM	694
Starch	g/kg DM	453
Calcium	g/kg DM	1.64
Phosphorus	g/kg DM	0.97
Magnesium	g/kg DM	0.62
Sodium	g/kg DM	0.25
Copper	mg/kg DM	5014
Zinc	mg/kg DM	9067
Selenium	mg/kg DM	<0.01

* Fatty acid pattern (%): 60 oleic/24 linoleic/14 palmitic/1.5 linolenic.

**Table 2:** Analysed nutrient composition and fatty acid profile of the two experimental diets.

Ingredients (g/kg Diet, as Fed)	Control Diet	Acorn Combined Diet
Alfalfa green meal	300	300
Wheat bran	270	270
Sunflower extruded meal	200	-
Hulled acorn shred	-	200
Oat peel bran	100	100
Barley	80	80
Beet pulp molasses	20	20
Calcium carbonate	19	19
Sodium chloride	6	6
Premix	5	5
Crude protein (g/kg DM)	160	146
Lysin (g/kg DM)	10.1	8.35
Met + Cys (g/kg DM)	9.34	7.19
Proline (g/kg DM)	13.0	10.4
Crude fibre (%)	170	162
Starch (g/kg DM)	236	241
Fatty acid composition (mg/kg of total fatty acids)		
14:0	0.69	0.68
14:1	0.17	0.14
16:0	28.1	17.7
16:1n-7	0.49	0.47
18:0	3.59	4.12
18:1n-9	28.1	22.5
18:2n-6	32.4	35.6
20:0	0.35	0.64
18:3n-3	5.82	15.4
20:1n-9	0.41	0.42
20:2	0.19	0.16
Σ SFA	32.77	23.1
Σ MUFA	29.2	23.5
Σ PUFA	38.4	51.2
Σ n-6/Σ n-3	5.57	2.31

**Table 3:** Production performance in rabbits (control vs. acorn combined diets) diet group (n = 20 rabbits/group; values expressed as means ± standard error).

Parameter	Control Diet	Acorn Combined Diet
Daily feed intake (g/d; days 37–76)	114 ± 26.4	122 ± 22.1
Feed conversion ratio (g/g)	3.19 ± 0.56	3.48 ± 0.31
Final body weight (g BM; day 76)	2130 ± 125	2157 ± 134
Salivary gland (% of BM)	0.252 ± 0.035 ^a^	0.325 ± 0.028 ^b^
Proline content (g/kg DM)	88 ± 30	93 ± 27
Liver weight (% of BM)	5.03 ± 0.78	5.15 ± 0.64
Perirenal fat (% of BM)	0.99 ± 0.17	0.95 ± 0.14
Carcass weight (g)	1394 ± 89	1402 ± 84
Cold carcass yield (%)	65.4 ± 2.57	64.9± 2.89

^a, b^ superscripts indicate significant differences for *p* < 0.05.

**Table 4:** Total lipid content and fatty acids composition of perirenal fat of rabbits fed with the two experimental diets (total lipids: g/kg; fatty acid composition: mg/kg fatty acids; mean ± SD).

	Control Diet	Acorn Combined Diet
Total lipids	73.6 ± 1.57	74.1 ± 1.83
14:00	2.41 ± 0.29	2.27 ± 0.45
14:01	0.14 ± 0.01	0.17 ± 0.08
16:00	31.8 ± 3.37	28.6 ± 2.49
16:1n-7	3.59 ± 1.74	2.71 ± 0.89
18:00	5.84 ± 0.19	6.21 ± 0.87
18:1n-9	30.5 ± 3.07	27.3 ± 2.21
18:2n-6	21.2 ± 0.64	22.1 ± 0.99
20:00	0.14 ± 0.08	0.15 ± 0.05
18:3n-3	3.30 ± 2.19	8.89 ± 4.89
20:1n-9	0.38 ± 0.03	0.37 ± 0.01
20:02	0.15 ± 0.00	0.16 ± 0.00
20:3n-3	0.11 ± 0.00	0.12 ± 0.01
20:4n-6	0.10 ± 0.01	0.14 ± 0.06
22:1n-9	0.06 ± 0.00	0.07 ± 0.01
20:5n-3	0.05 ± 0.00	0.06 ± 0.01
24:00:00	0.06 ± 0.01	0.05 ± 0.00
24:1n-9	0.07 ± 0.01	0.04 ± 0.00
22:5n-3	0.06 ± 0.01	0.05 ± 0.00
22:6n-3	0.06 ± 0.01	0.09 ± 0.02
Σ SFA	40.3	37.3
Σ MUFA	34.7	30.7
Σ PUFA	25.1 ^a^	31.6 ^b^
Σ n-6/Σ n-3	5.95 ^a^	2.41 ^b^

^a, b^ superscripts indicate significant differences for *p* < 0.05.
